# Some Aspects of Suicide

**Published:** 1893-08

**Authors:** William H. Palmer

**Affiliations:** Providence, R. I.


### HALL'S JOURNAL OF HEALTH

HEALTH—THE POOR MAN'S RICHES, THE RICH MAN'S BLISS.

VOL. XL. AUGUST, 1893. No. 8.

### SOME ASPECTS OF SUICIDE.

Suicide is commonly regarded as either an insane act, a moral offence, or, from a sensational standpoint, as an heroic achievement. Besides these aspects there are others resulting from the consideration of suicide as the direct and evident outcome of given social conditions.

Self-destruction is sometimes, though not usually, the result of insanity. In Europe the proportion of suicides to the insane has been estimated by competent observers as about one-third. In this country sufficient statistics to demonstrate the proportion of suicides to the admittedly insane have not been collected, but this proportion is doubtless smaller than is popularly supposed. In most cases of suicide there is evidence of deliberate rational preparation for the act. The suicide is incited by real motives arising from actual facts, and in no way based upon hallucinations. Instances are many where it is possible to show that the suicide has brought his reasoning powers to bear upon this act as well as upon any other act of his life. Consideration for the feelings of friends often covers a case of suicide under the cloak of "temporary mental aberration," when in reality the deed has been well pondered and sanely accomplished. Attempts to defraud life insurance companies by suicide after obtaining heavy insurance for the benefit of a family, have been contrived and carried out as ingeniously as bank robberies, with the expectation of success resting upon the belief that the average juryman will regard the act of suicide as in itself an evidence of insanity. And the expectation is well founded, notwithstanding the fact that both state and national courts have established the opinion that there is no presumption in law that self-destruction arises from insanity.

To encourage the supposition that the suicide is probably insane, is a mistake which may possibly lend itself to fraud, and which certainly diminishes the means whereby the prevention of suicide may be sought.

On the other hand to relegate suicide to the category of moral crimes is to absolve society from responsibility, leaving the decision for or against the act to the conscience of the actor. Hamlet wished that

> "The Almighty had not fixed
> His canons 'gainst self-slaughter,"

but believing them to be so fixed he did not attempt to justify himself for violating them. But conscience is less alert in this era than in Hamlet's day, certainly the consciences of those "rashly importunate" beings who fly from the ills they have, to others that they know not of, by suicide. Neither is suicide to be justified by the social ethics of our day as it was by a pagan philosopher, and the correction of a misplaced admiration for the heroic element in the deed, is a means to be employed toward checking it.

Suicide is a crime against society, the result of an exaggerated idea of individual independence, and of extra sensitiveness to failure, to disgrace, and to disappointments in life. Its history is interesting from the fact that some of the greatest minds the world has produced, have justified the resort to suicide, not only in theory but in practice. Themistocles killed himself rather than lead the Persians against his country men. Marc Antony died because he believed Cleopatra false to him; Aristotle wrote "Courage is the mean between fear and rashness, while suicide is the sane of both," and then took his own life. The physician who attended the Princess Charlotte in her fatal confinement (Sir R. Croft) committed suicide because his professional reputation was blasted, and Roubeau, a young French doctor, took his life because of his mortification at the reception of a medico-philosophic work he had published.

Suicide in times past as well as at present, may be said to have been a popular manner of ending an unsatisfactory career, of showing resentment at failure and remorse for crime.

Suicide has doubtless been contemplated by many a great mind which has nevertheless refrained from the act. Shakespeare's early sonnets, as well as his subtle examination of "to be or not to be" in the play, indicate how intimately his thought had dallied with the subject. Goethe in his autobiography frankly records the manner in which he had weighed the notion of closing the fifth act of his tragedy according to his choice, because of the "immoderate demands which life made upon him."

Suicide is undoubtedly on the increase at the present time. Few daily papers in the large cities but record at least one instance of self-destruction in the community. Dr. Hamilton estimates the increase of suicide at the rate of 300 per cent. every seven years. At the date of this writing these items were clipped from a single issue of a daily paper. They are severally headed ;

(1) *Charles S. Rogers'* (President of the Northwestern Cordage Works) *body found.* He committed suicide May 19th, by drowning in the Mississippi river. Cause, business troubles.

(2) Double tragedy in an Indiana town. Christopher Haberkus killed his wife and then himself by cutting his throat. Cause, domestic disagreement.

(3) *Tired of life,* " *nothing to live for.*" Robert Proctor of Leominster, Mass.

(4) *A suspicious death.* Supposed suicide by shooting, of the city editor of the *Staats Zeitung*, New York city, after a quarrel with his wife.

(5) Suicide in a Chicago hotel, of F. H. Milburn, son of the blind Chaplain of the U. S. House of Representatives. Cause, penniless and laboring under great despondency.

These daily occurrences brought to our attention, thrust certain inquiries upon us regarding an act which contradicts the strongest instincts of human nature ; such inquiries as—What are the characteristics of suicide? What manner of men and women resort to it? What are the chief motives leading to it? And what can be done to prevent it?

The annals of suicide reveal some constant characteristics, such as (1) its increased frequency at certain seasons of the year. This increased frequency is during the early spring and summer, reaching its maximum in June. (2) The prevalence of the suicidal impulse in the vicinity of great rivers as compared with mountainous regions. (3) The force of example manifested in suicides which are the result of imitation. The case of Colburn's leap from the Brooklyn bridge is to the point. It will be remembered that constant vigilance on the part of the police was for a time required to prevent others from following his example ; and in Paris, the ascent of the Vendome column has been interdicted because so many sought death by throwing themselves from it—following the fashion.

There is probably no such thing as a suicidal temperament. All sorts and conditions of people resort to it. About two-thirds of the suicides are from the working class, the remainder from among the well-to-do, the literary and professional classes. The number of men suicides is considerably in excess of that of women. Among nations, the modern Germans, the most profound and philosophic thinkers of this time, are also the most suicidal race in Europe. Between the northeast of France and the eastern borders of Germany is an area called "the classic ground of suicide." Out of 359 suicides during three years (1870–71–72) in New York city, 132 were Germans.

The most urgent motive to suicide is misery, from one cause or another.

The civilization of our day despises humble attainments and simple pleasures. There is a race for luxury and renown, for higher and higher successes, both intellectual and material. The struggle is intense, failure unbearable. The result is the razor to the throat, the bullet to the brain. We live fast and the suicide is usually but another unfitted to keep step. Though not insane, he is handicapped by inertia, which hinders his will from reacting against adverse fate. Despondent, reckless and contemptuous, he seeks to end it all. The drunkard may commit murder under the frenzied excitement which renders him for the moment insane, but he takes his own life when the debauch is ended and reason has reasserted her sway. It is well considered discontent with life that we find the paramount cause for voluntarily quitting it. And in proportion as the conditions of life are unfavorable to an approximate success, we shall find the motives for self-destruction strong and suicides frequent. Among the poor, crowded in unwholesome tenements, working in mills and shops under bad sanitary conditions, vices are engendered by the surroundings, which lower the vital tone and tend to discouragement and suicide. Among these vices, seduction and sins against chastity are prolific sources of misery. They destroy self-respect and thereby decrease the love of life, the power of endurance, the hope for better things, and are instrumental in inciting to suicide.

In the depraved mental and physical conditions which we find among bad surroundings, prompting to self-destruction, we come, it is true, into the border land between sanity and insanity. But diverse propensities, variations in strength and weakness of will, are not to be characterized as insanity. For insanity there must be a differentiation from the normal mental states and moral habits, a change from the habitual point of view—in short derangement. The modification of a customary mood is not insanity. Two-thirds of the suicides committed are among the poor. Many of these would be prevented by the amelioration of their surrounding conditions, by better food, light, air, and by the inspiration of hope.

Education is to be relied on as a potent force for the prevention of suicide, especially that form of education which has as its chief object the formation of a strong character and a stable will.

Wholesome amusements in place of lewd entertainments tend to a frame of mind into which thoughts of suicide do not enter; while the literature which presents distorted pictures of life, suggest base forms of enjoyment, over-stimulates the emotional nature, and encourages expectations impossible to realize, feeds dissatisfaction with the actual, and makes it appear that life is not worth living.

The law already recognizes, as it should, the criminality of suicide, and the sale of poisonous drugs should always be regulated by legislation; but the prevention of two-thirds of the suicides which occur is mainly to be effected through social amelioration and education.

As for the remaining one-third of the suicide class, the well-to-do and well endowed, those who have no right to complain that they have not had a fair chance in the struggle for existence, the means of prevention of suicide among this class must be found in the strengthening of the sense of social responsibility, and in maintaining the position that suicide is a social crime. Society is an association for a struggle with nature and with the adverse, anti-social elements. The struggle requires a united effort. Progress depends upon the discipline and self-control of unbroken ranks. The suicide is the deserter from these ranks, who thinks only of his own discomfiture, who

> "Flings his own load off,
> And leaves his fellows toiling."

The physician's personal opinion regarding suicide may, on occasion, become a means of preventing the act of self-destruction and the turning point whereon a life hinges. When a patient inquires his physician's opinion of this subject, he is pretty sure to have some thought of the deed in his mind. The medical man needs to be prepared to make plain the craven nature of the deed, and to have at his tongue's end the argument to prove that the suicide is the shirk.

Rather than by legislative enactments the prevention of suicide must be sought by diminishing the causes of misery and despondency and by heightening in each the sense of his individual value and responsibility.

Providence, R. I. WM. H. PALMER, M. D.

